# Synthesis of Chiral MOF‐74 Frameworks by Post‐Synthetic Modification by Using an Amino Acid

**DOI:** 10.1002/chem.202002293

**Published:** 2020-10-20

**Authors:** Andreea Gheorghe, Benjamin Strudwick, Daniel M. Dawson, Sharon E. Ashbrook, Sander Woutersen, David Dubbeldam, Stefania Tanase

**Affiliations:** ^1^ Van ‘t Hoff Institute for Molecular Sciences University of Amsterdam Science Park 904 1098 XH Amsterdam The Netherlands; ^2^ Current address: Paul Scherrer Institute ETH Zürich Forschungsstrasse 111 5232 Villigen PSI Zürich Switzerland; ^3^ EaStCHEM School of Chemistry and Centre of Magnetic Resonance University of St Andrews North Haugh KY16 9ST St. Andrews UK

**Keywords:** chiral induction, chirality, defects, metal–organic frameworks, post-synthetic modifications, vibrational circular dichroism

## Abstract

The synthesis of chiral metal–organic frameworks (MOFs) is highly relevant for asymmetric heterogenous catalysis, yet very challenging. Chiral MOFs with MOF‐74 topology were synthesised by using post‐synthetic modification with proline. Vibrational circular dichroism studies demonstrate that proline is the source of chirality. The solvents used in the synthesis play a key role in tuning the loading of proline and its interaction with the MOF‐74 framework. In *N*,*N*′‐dimethylformamide, proline coordinates monodentate to the Zn^2+^ ions within the MOF‐74 framework, whereas it is only weakly bound to the framework when using methanol as solvent. Introducing chirality within the MOF‐74 framework also leads to the formation of defects, with both the organic linker and metal ions missing from the framework. The formation of defects combined with the coordination of DMF and proline within the framework leads to a pore blocking effect. This is confirmed by adsorption studies and testing of the chiral MOFs in the asymmetric aldol reaction between acetone and *para*‐nitrobenzaldehyde.

## Graphical abstract

**Chiral MOF‐74 materials** were synthesised by post‐synthetic modification by using proline as chiral inductor. Combined experimental and theoretical studies show that proline coordinates monodentate to the Zn^2+^ within the metal–organic framework (MOF). Along with introducing chiral features, defects are formed in the MOF‐74 topology.

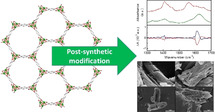

## Introduction

Chirality plays a key role in the understanding of biochemistry[^1^, ^2^] and it remains a long‐standing enigma for explaining the origin of life because all chiral amino acids in proteins and enzymes exist only in their l form.^[3]^ Producing chiral compounds is essential for the pharmaceutical,^[4]^ food,[^5^, ^6^] agricultural^[7]^ and biotechnological industries.^[8]^ For the pharmaceutical industry, the activity of a drug is based on its specific interaction with the target made up of chiral fragments (e.g., proteins, nucleic acids, etc.).^[9]^ Thus, the biological and pharmacological influence of the drug is highly dependent on its chiral form.^[9]^ The synthesis strategies of chiral molecules include chemical transformation of enantiopure precursors from the chiral pool,^[10]^ racemic resolution,^[11]^ but also asymmetric synthesis.^[12]^ Efficient homogeneous enantioselective catalysts are currently used in asymmetric synthesis, but there are still challenges related to the catalyst sensitivity to impurities, catalyst or ligand stability, the requirement of extreme conditions, as well as the unavailability of the ligand or the catalyst.[^13^, ^14^, ^15^] Alternatively, the use of heterogeneous catalytic systems enables the easy recovery of the catalyst from the reaction medium and its reuse in consecutive cycles, therefore resulting in low‐cost applications. In this context, chiral metal–organic frameworks (MOFs)^[16]^ have emerged as a new class of potential asymmetric heterogeneous catalyst.[^17^, ^18^, ^19^, ^20^]

A straightforward approach towards the synthesis of chiral MOFs relies on the appropriate combination of metal ions and enantiopure organic linkers.[^18^, ^21^, ^22^, ^23^, ^24^, ^25^] Although some efficient catalysts are designed by using this approach, the synthesis of the chiral linkers often entails lengthy multi‐step procedures. An alternative methodology focuses on the post‐synthetic modification of achiral MOFs by either covalent binding of chiral groups on the organic linkers^[26]^ or by coordinating them to the metal ions.^[27]^ For example, the achiral Cr‐MIL‐101 framework, namely [Cr_3_O(H_2_O)F(bdc)_3_]**⋅**4.5 H_2_O⋅0.15(H_2_bdc) where H_2_bdc=terephthalic acid, was converted to the isostructural chiral forms (CMIL‐1 and CMIL‐2) by using a proline derivative ligand, which is post‐synthetically coordinated to the metal centres.^[27]^ Recent studies also demonstrated the chirality transfer between chiral molecules and MOF crystallites through weak or strong intermolecular interactions.^[28]^ This synthetic strategy is known as chiral induction and can be achieved by using chiral templates, additives or solvents.[^28^, ^29^, ^30^, ^31^, ^32^]

Considering the structural variety of MOFs currently available,^[20]^ a straightforward synthetic approach for making chiral MOFs would imply the induction of chirality in existing network topologies. Using this synthetic strategy, a chiral MOF‐5 topology was prepared via the direct synthesis of MOF‐5 in the presence of l‐ or d‐proline and by using *N*‐methylpyrrolidone (NMP) as solvent.^[33]^ In the presence of l‐proline, Λ‐CMOF‐5 is formed, whilst the addition of d‐proline lead to Δ‐CMOF‐5.^[33]^ The single‐crystal XRD analysis of both structures shows that the benzene rings between adjacent Zn_4_O clusters are oriented at a non‐90° dihedral angle (78° for Λ‐CMOF‐5 and 66° for Δ‐CMOF‐5) and the atropisomer‐like bridging mode of the benzene‐1,4‐dicarboxylate linkers translates the chirality from one cluster to another.^[33]^ Moreover, the integrity and chirality of the structures was preserved due to the stabilising effect of the achiral NMP solvent molecules.^[34]^ Similarly, the chiral induction was achieved in (Me_2_NH_2_)[In(thb)_2_]**⋅** *x* DMF (H_2_thb=thiophene‐2,5‐dicarboxylic acid) using chiral alkaloids.^[35]^ In another study, the synthesis of chiral [Mn_3_(HCOO)(d‐cam)] or [Mn_3_(HCOO)(l‐cam)] was attained via the coordination of the chiral additive, d‐ or l‐camphoric acid (d‐ or l‐Hcam), to the metal sites of the framework.^[31]^

The studies so far indicate that the chiral induction during synthesis might indeed be a facile and inexpensive approach to synthesise chiral MOFs. It also shows that the chemical interactions between chiral additives and the MOF framework is a key parameter. However, the examples above also illustrate that the chemical functionality of the chiral inductor matches well the chemical functionalities of the organic linkers, for example, carboxylate groups. The challenge still lies in the ability to select the proper chiral additive that enables the chiral transfer while retaining the framework topology. For instance, in the formation of CMOF‐5, the chiral proline inductor determines the handedness, but the chirality stems from and is stabilised by the NMP guest molecules.[^33^, ^34^] This achiral solvent used for synthesis creates an internal stress on the framework and twisting of the organic linkers that lead to a chiral framework.^[34]^

**Figure 1:**
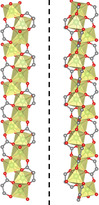
The left‐ (*M* or *L*) and right‐handed (*P* or *D*) helical SBU rods of Zn‐MOF‐74.

The aim of this work was to study and to understand if the chiral induction can be applied to other structural topologies and to gain further insight on the parameters governing the chiral induction process. It is focused on a material, which is recognised as the MOF with the highest density of open metal sites, namely [Zn_2_(dobdc)(H_2_O)_2_] (H_4_dobdc=2,5‐dioxido‐1,4‐benzenedicarboxylic acid), known as Zn‐MOF‐74.^[36]^ Zn‐MOF‐74 is intensively studied for gas storage^[36]^ and separation^[37]^ as well as in catalysis.[^38^, ^39^, ^40^, ^41^, ^42^, ^43^, ^44^, ^45^] It has a 3D honeycomb‐like framework with 1D channels of ca. 11 Å,^[46]^ built from organic linkers on the edges and ZnO_6_ at the corners. The ZnO_6_ octahedra form infinite helical chains via double oxygen bridges along the *c* axis, but of opposite handedness with *P* and *M* helical rods with a ratio *P*/*M* of 1:1 (Figure 1). In recent work, we showed that by using chiral cinchona alkaloids during the crystallisation of Zn‐MOF‐74 does not influence the *P*/*M* ratio or induce chirality. The bulkiness of the chiral molecule and its weak coordination to the metal ions afforded achiral structural isomers with discrete secondary building units (SBUs).^[46]^ Consequently, we turn our attention to chiral inductors which are smaller and would easily be accommodated in the channels of Zn‐MOF‐74, for example, the chiral amino acid l‐ or d‐proline (l‐ or d‐Pro). Here, we discuss the synthesis and characterisation of the chiral materials obtained by using chiral induction with l‐ or d‐Pro.

## Results and Discussion

The first approach to synthesise a chiral Zn‐MOF‐74 topology aimed at using d‐ and l‐Pro as chiral inductor during the typical synthesis of Zn‐MOF‐74.^[47]^ This molecule has the ability to interact with the MOF framework via hydrogen bonding with the carboxylate groups of the organic linkers or through coordination to the open sites on the Zn^2+^ ions.^[48]^ The size of d‐ or l‐Pro molecules is ca. 8.5×5.6×5.3 Å^3^, indicating that they can enter within the MOF‐74’s channels of ca. 11 Å. Therefore, we hypothesised that the interaction of l‐ or d‐Pro with Zn‐MOF‐74 may lead to a chiral framework and that proline, as auxiliary ligand, will lead to a long‐range chirality order. Earlier studies have shown that using l‐Pro as modulator in the synthesis of Zr^4+^ and Hf^4+^ MOFs with UIO‐66 topology affords access to large and high quality single‐crystals that are free of defects^[49]^ and that l‐Pro can be incorporated as a defect‐capping structural element.[^50^, ^51^] Recently, the chirality of l‐Pro modulated Zr^4+^‐based MOFs was demonstrated in the aldol reaction between 4‐nitrobenzaldehyde and cyclohexanone.^[52]^

Our group reported the synthesis of MOF‐74 in the presence of bulky chiral alkaloids leading to a new structural topology, HIMS‐74, which is built from binuclear secondary building units.^[46]^ We attributed the formation of the binuclear SBUs to the N,O‐coordination of the alkaloids to the Zn^2+^ ions during the nucleation process, thereby inhibiting the formation of the chain‐like SBUs, which are characteristic of the MOF‐74 topology. In subsequent studies, we replaced the bulky chiral alkaloids with the smaller proline molecule. Our hypothesis was that, unlike the reactions performed by using chiral alkaloids, the use of d‐ or l‐Pro during the synthesis of MOF‐74 would lead to chiral materials because of the reduced size of the additive. Attempts to crystallise a chiral MOF‐74 topology with l‐Pro via induction were unsuccessful, even when varying the synthetic parameters (molar ratio, solvent(s), temperature and scale of the reaction) or when using other amino acids (l‐valine, l‐alanine, l‐tryptophan). The hydrothermal synthesis of MOF‐74 in the presence of amino acids yielded powders that were amorphous as indicated by PXRD studies. This could be due to the low solubility of the amino acids in the reaction mixture (DMF, NMP/H_2_O, DMF/H_2_O/EtOH). In the functionalisation of Zr‐UIO‐66 and Zr‐UIO‐67 MOF with l‐Pro, HCl was added to ensure the dissolution of l‐Pro by formation of hydrochloride salts of l‐Pro that allowed the growth of MOF structures.^[51]^ However, performing the crystallisation of MOF‐74 in the presence of l‐Pro and HCl did not lead to any solid material, indicating that the presence of the acid inhibits the framework formation.

**Figure 2:**
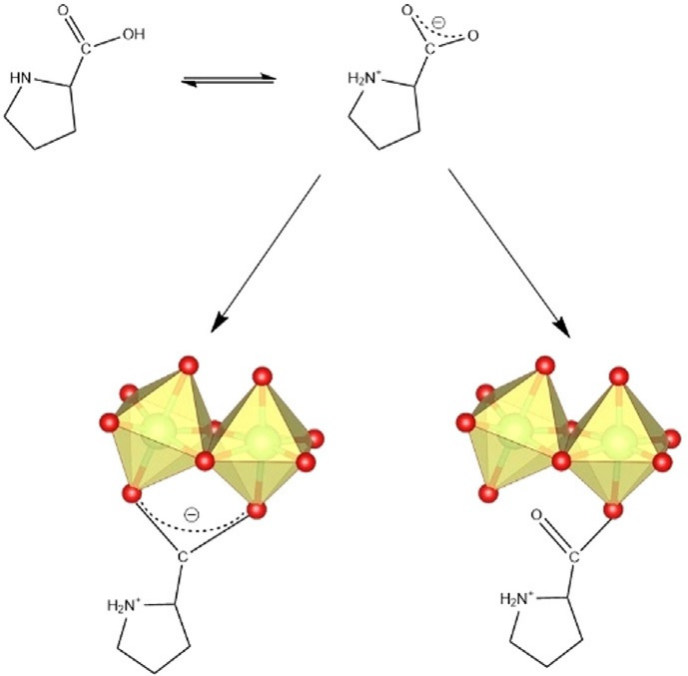
The tautomeric species of l‐ or d‐Pro and their expected coordination to the SBUs of MOF‐74.

To overcome the challenges discussed above, we focused on a second approach based on the post synthetic chiral induction of Zn‐MOF‐74. The material used was prepared by using a water‐based room‐temperature synthesis. Solvent exchange by using methanol (MeOH) and subsequent vacuum heating were carried out prior to the post synthetic modification in order to obtain the Zn‐MOF‐74 material containing open metal sites. Considering that the molecular size of d,l‐Pro enables its diffusion within the pores of Zn‐MOF‐74, we anticipated that the chiral amino acid might coordinate to the Zn^2+^ ions in a monodentate manner via its carboxylate group or bidentate, bridging neighbouring Zn^2+^ ions while maintaining the six‐fold coordination of the Zn^2+^ ions (Figure 2). Another expected interaction is the intermolecular hydrogen‐bonding of d,l‐Pro with the carboxylate groups of the organic linker.

The reaction of d,l‐Pro with Zn‐MOF‐74 was performed in two solvents of different polarities: *N*,*N*′‐dimethylformamide (DMF) commonly used when synthesising MOF‐74 materials, and MeOH that is usually employed for solvent exchange. We hypothesised that DMF, being a strongly coordinated solvent, may compete with the coordination of d,l‐Pro to the Zn^2+^ metal ions more than MeOH, which is known to have less binding strength towards metal ions as compared to DMF. This was confirmed by calculating the binding energy values for MeOH, DMF, and proline to the Zn^2+^ ions of a Zn‐MOF‐74 unit cell. Indeed, the binding energies for both proline and DMF were lower than the one for MeOH (−150.78 kJ mol^−1^). Three different binding energies of l‐Pro were calculated when considering all possible interactions of l‐Pro with the framework. For proline molecules retained in the pore via weak interactions, an energy of −202.37 kJ mol^−1^ was calculated, slightly higher than the DMF binding energy of −216.12 kJ mol^−1^. The energies corresponding to proline coordinating to the metal ions are significantly higher, both for bidentate (−341.55 kJ mol^−1^) and monodentate (−468.46 kJ mol^−1^) coordination.

**Figure 3:**
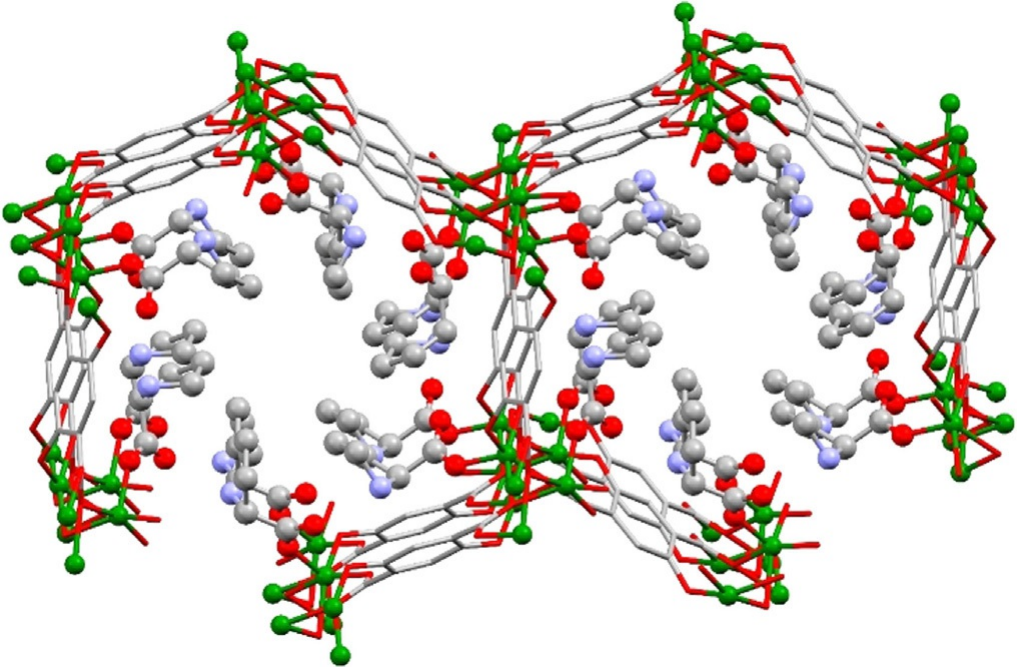
View along the *c* axis of the cell of Zn‐MOF‐74 fully loaded with proline coordinated in a monodentate manner.

**Figure 4:**
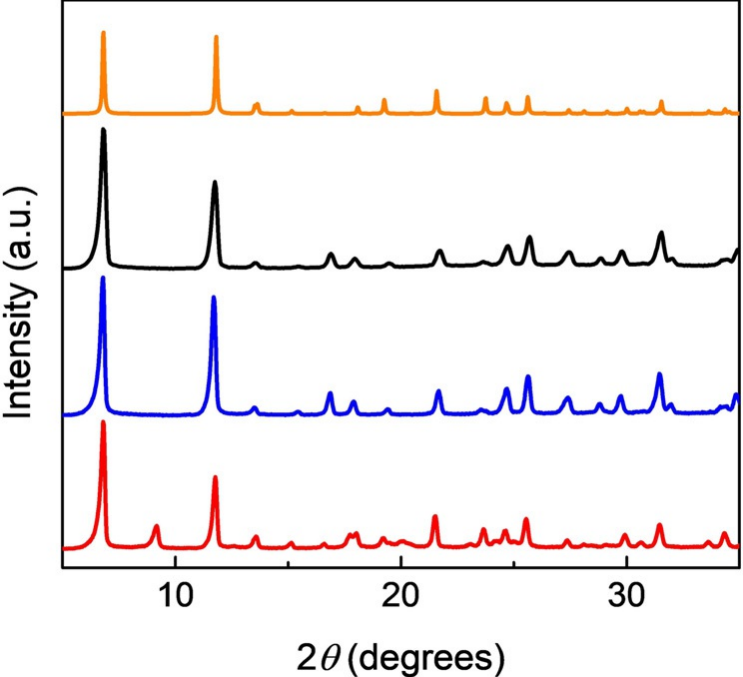
PXRD patterns of the as‐synthesised Zn‐MOF‐74 (black) and Zn‐MOF‐74‐l‐Pro synthesised in MeOH (blue) and DMF (red). The calculated PXRD pattern of Zn‐MOF‐74 (orange) was obtained from literature.^[53]^.

In a typical synthesis, Zn‐MOF‐74 was suspended in a solution containing the d‐ or l‐Pro in a molar ratio MOF to d,l‐Pro of 1:1 and stirred at ambient conditions for 48 h. The corresponding amount of proline would fill up to a half the unit cell of MOF‐74, with a formula of Zn_2_(dobdc)(Pro) based on a fully loaded MOF with proline coordinated monodentate to the Zn^2+^ ions via the carboxylate oxygen (Figure 3). This was chosen in view of its potential application, such as asymmetric catalysis in which the diffusion within the MOF channels is a key parameter. Thus, we choose the Zn^2+^ to Pro molar ratio of 2:1 to prevent possible pore blocking. The recovered solid materials were analysed using complementary techniques to determine their purity, stability and chirality. There is a perfect match between the PXRD patterns of Zn‐MOF‐74 and its derivatives prepared in MeOH and in the presence of l‐Pro, indicating that the overall framework topology is retained (Figure 4). For the materials prepared in DMF, the PXRD pattern reveals a new peak around 2 *θ*=9.2° that indicates a change in the framework. Notably, the PXRD analysis also shows that the type of compounds obtained in MeOH and DMF are the same, irrespective of the nature of the enantiomer used (see Figure S1 in the Supporting Information for details).

**Figure 5:**
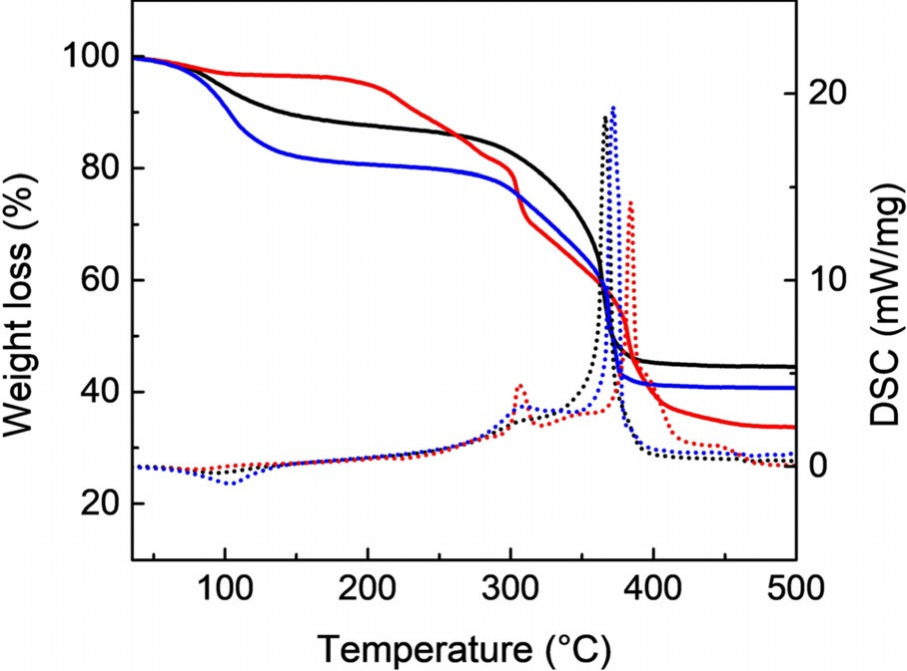
TGA (continuous line) and DSC (dotted line) curves of Zn‐MOF‐74 (black) and Zn‐MOF‐74‐l‐Pro synthesised in MeOH (blue) and DMF (red).

Thermogravimetric analysis (TGA) combined with differential scanning calorimetry (DSC) was carried out on the modified MOFs and MOF‐74 sample (Figures 5 and S2). The Zn‐MOF‐74 was characterised by TGA‐DSC and it shows an initial ca. 14 % weight loss at around 100 °C corresponding to H_2_O molecules as a result of the air exposure after activation. The decomposition of the framework is indicated by the sharp exothermic peak observed in the DSC at ca. 365 °C (Figure 5). Synthesis of Zn‐MOF‐74‐l‐Pro in MeOH shows a much larger initial weight loss of ca. 21 % accompanied by a broad DSC peak around 100 °C that can be assigned to solvent molecules, both coordinated and adsorbed in the material. Above 100 °C, both TGA and DSC curves are similar to those obtained for the Zn‐MOF‐74. The Zn‐MOF‐74‐l‐Pro sample prepared in DMF shows a relatively stable plateau until ca. 200 °C, as it is expected for the strong interactions established between DMF and the Zn‐MOF‐74 framework. The framework decomposition is slightly shifted to a higher temperature as compared to the pristine material, also in agreement with the PXRD data that indicate that some structural changes occur. Similar results are observed for the Zn‐MOF‐74‐d‐Pro prepared in MeOH and DMF (Figure S2).

An uptake of ca. 1.2 % l‐Pro was calculated for the sample prepared in MeOH, based on the DTG curve related to the broad peak in the region 260–325 °C, which corresponds to the removal of l‐Pro from the framework (Figure S3). This indicates that l‐Pro is adsorbed and possibly bound through weak interactions in the Zn‐MOF‐74 framework. For the material prepared in DMF by using l‐Pro, an uptake of ca. 6.9 % was also calculated using the DTG curve (Figure S3). In this case, the sharp exothermic peak at 306 °C in the DSC curve indicates a monodentate coordination of l‐Pro to Zn^2+^, via one oxygen from the carboxylate group (Figure 5).^[54]^ The bidentate coordination of l‐Pro to the Zn^2+^ ion is unlikely, as it would give a DSC peak at higher temperature, ca. 345 °C, in agreement with a stronger binding effect.^[55]^

FTIR spectra were recorded to further prove the presence of the l‐ and d‐Pro within the framework of the synthesised materials. Similar to the PXRD studies, independent of the handedness of the chiral amino acid, the materials have identical FTIR spectra (Figures S4 and S5). The higher concentration of l‐ and d‐Pro in the materials obtained in DMF allows to identify the characteristic vibration modes, including the NH stretching at 3216 cm^−1^ and the CH_2_ vibrations in the range 2800–3200 cm^−1^ (Figure S5).^[55]^

To probe the existence of chirality within the synthesised materials, we used the vibrational circular dichroism (VCD) technique. VCD measures the differential absorption of left‐ and right‐handed circularly polarised infrared light^[56]^ and it is a powerful spectroscopic method to analyse the chirality at the molecular level. VCD is a technique that extends the regular circular dichroism (CD) measurements into the infrared region.^[43]^ It is a very simple spectroscopic measurement and can be used to obtain chiral information for molecules in all phases. Considering that the VCD signals originate from vibrational transitions, it can be used to study all molecules, even if they lack an UV chromophore as needed for CD.^[57]^ So far, it has been used to investigate small molecules,^[38]^ synthetic polymers,^[39]^ but also metal coordination polymers.[^40^, ^41^, ^42^, ^43^] Furthermore, the quality of the VCD spectroscopic data is highly related to the sample homogeneity,^[58]^ therefore our studies focused on post‐modified materials synthesised using a Zn‐MOF‐74, which is highly homogeneous and has crystals with narrow size distribution.^[46]^

**Figure 6:**
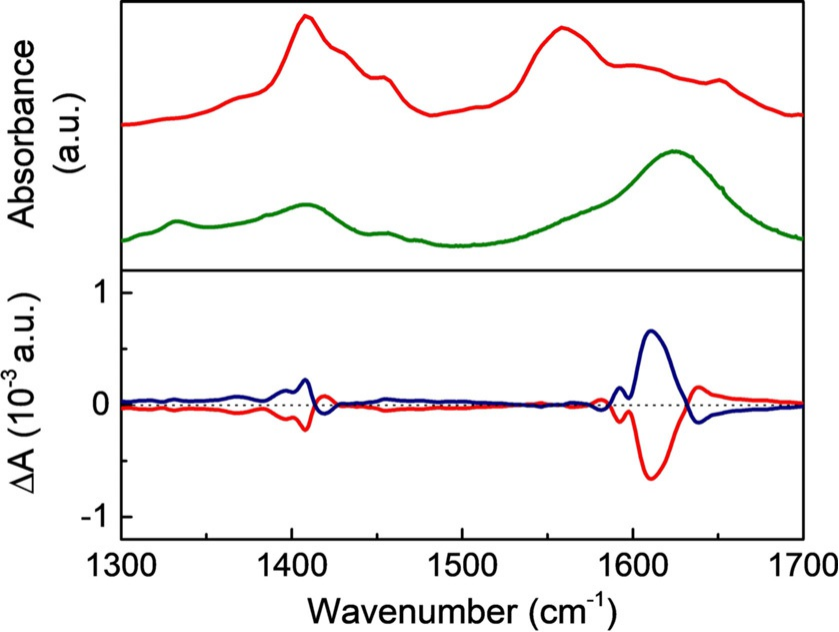
FTIR spectra of l‐Pro (green) and Zn‐MOF‐74‐l‐Pro (red) (top) and VCD spectra of the Zn‐MOF‐74‐l‐Pro (red) and Zn‐MOF‐74‐d‐Pro (blue) (bottom), all MOF materials being synthesised in DMF.

The samples prepared in DMF show strong VCD signals in the region 1300–1700 cm^−1^ assigned to the coordinated proline molecules. The signal at 1609 cm^−1^ correlates very well with the IR band at 1600 cm^−1^. This band is assigned to the asymmetric stretching of the carboxylate group of proline (Figure 6).^[59]^ Moreover, there is a band shift to a lower wavenumber when compared to the free proline, ν_as_(COO^−^)=1626 cm^−1^, in agreement with the coordination of the carboxylate group to the Zn^2+^ ions.^[59]^

This also leads to a shift in the position of the VCD signals (Figure 6) as compared the VCD spectra of l‐ and d‐Pro (Figure S6). The presence of opposite signals originating from Zn‐MOF‐74‐l‐Pro and Zn‐MOF‐74‐d‐Pro indicates that the amino acid retains its chirality upon coordination to the framework. For the samples prepared in MeOH, the signals are relatively small when compared to those synthesised in DMF (Figure S6). This correlates to the very low content of amino acid present in the MOFs. The weak VCD signals for the samples prepared in MeOH match the ones of l‐ and d‐Pro (Figure S6) because proline is not coordinated to the metal ions as indicated by the broad peak around 300 °C in DSC (Figure 5).

**Figure 7:**
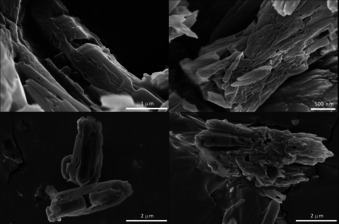
SEM images of Zn‐MOF‐74‐l‐Pro (top) and Zn‐MOF‐74‐d‐Pro (bottom).

For the materials synthesised in DMF, TGA and DSC analysis indicate a monodentate coordination of proline via one oxygen of the carboxylate group. Computational methods were used to determine the most favourable coordination of proline to the Zn^2+^ ions within the framework of Zn‐MOF‐74‐l‐Pro, which indeed strongly supports the monodentate binding. The calculations also show that the bidentate coordination of proline to the open sites of two neighbouring Zn^2+^ ions would cause a strain on the framework that, in turn, would lead to a twisting of the linker molecule in order to accommodate the coordinated proline molecule (Figure S7). This is a much less stable framework than the initial one. Although the monodentate coordination of proline to Zn^2+^ is nicely demonstrated by both experimental and theoretical studies, the PXRD analysis indicates clearly that structural changes have occurred. The calculated PXRD pattern, when adding metal ions in the channels of MOF‐74, indicates that the peak observed at 2 *θ*=9.2° corresponds to the presence of Zn^2+^ ions in the middle of the hexagonal channels of the Zn‐MOF‐74 framework. It suggests that defects are formed during the post‐synthetic modification of Zn‐MOF‐74 and some Zn^2+^ ions have migrated within the framework. This means that the post‐synthetic modification of the MOF in DMF has led to the formation of defects. By using ^1^H NMR analysis of the digested MOFs, the ratio of ca. 1:1.7 linker to l‐Pro was calculated for the sample prepared in DMF (Figure S8). This was not possible for the samples synthesised in MeOH most likely due to the low amount of amino acid. Since the initial 1:1 ratio of linker to amino acid is changed significantly, it is clear that the post‐synthetically modified MOFs have missing linker defects due to the dissolution of the MOF during the loading process. This was further observed when analysing the sample morphology as SEM images show that the integrity of the crystals is heavily damaged (Figure 7).

To further confirm the coordination of proline and to investigate the structural changes observed, solid‐state ^13^C CP MAS NMR studies were carried out for the samples prepared by using l‐Pro in DMF and MeOH. The ^13^C CP MAS NMR spectra of Zn‐MOF‐74‐l‐Pro obtained in MeOH (Figure S9) shows four resonances at 174.0, 156.8, 127.7 and 126.5 ppm, corresponding, to C1, C3, C4 and C2 of the dobdc^4−^ linker, respectively. In line with the TGA‐DSC data, there is no methanol signal (expected at ≈50 ppm) and, due to the low amount of amino acid, there were no l‐Pro signals. This is not the case for Zn‐MOF‐74‐l‐Pro synthesised in DMF that exhibits slightly broader resonances for the organic linker (173.9, 157.5, 127.4 and 126.8 ppm), as well as a broad resonance at 165.7 ppm assigned to DMF. The C3 peak of the linker is most affected by guest adsorption, suggesting perhaps an NH⋅⋅⋅O(C3) hydrogen bonding interaction between proline and the MOF or a distortion of the coordination environment of the Zn so that the Zn−O(C3) bond is lengthened. The remaining signals can be attributed to l‐Pro and the two inequivalent CH_3_ groups of DMF. The carboxylate region contains two signals for C6 of l‐Pro at 181.3 and 178.7 ppm, suggesting two molecular conformations that can only be due to the partial racemisation of l‐Pro. This was not expected due to the mild synthesis conditions used and racemisation of l‐Pro upon PSM was previously reported only for the post‐synthetic thermal deprotection of DUT‐32‐NHPro.^[60]^ The other resonances for l‐Pro are also split with the C2 signal at 62.0 and 60.5 ppm, C5 at 50.2 and 48.1 ppm, and C3 and C4 at 37.9, 33.7, 31.4, 29.6 and 28.7 ppm.

**Figure 8:**
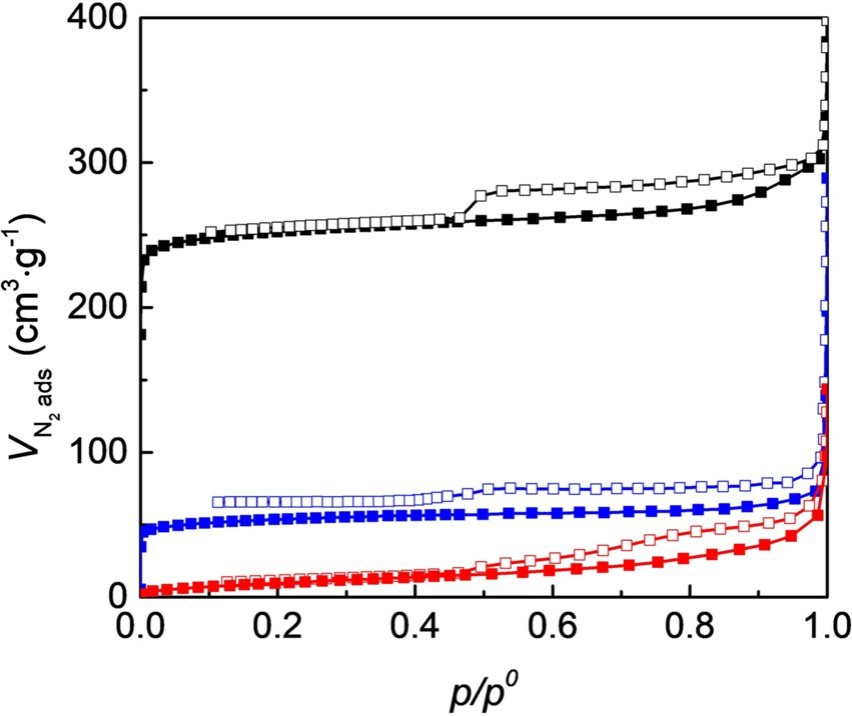
N_2_ adsorption isotherms measured at 77 K for Zn‐MOF‐74 (orange), Zn‐MOF‐74‐l‐Pro synthesised in MeOH (blue) and Zn‐MOF‐74‐l‐Pro synthesised in DMF (red).

Considering that the chiral Zn‐MOF‐74 materials are highly relevant for applications in asymmetric catalysis or enantioselective recognition, the porosity of the materials was studied using nitrogen adsorption (Figure 8). The results show that proline is preferentially introduced in the framework, resulting in a decrease of the nitrogen adsorption. This was also observed in previous reports for proline‐modulated Zr‐MOFs.[^47^, ^52^, ^61^] For DUT‐67‐Pro, the surface area is decreased after proline binds to the Zr‐cluster by solvent assisted ligand incorporation (SALI), exchanging a posteri the acetate ions.^[52]^ Notably, for the Zn‐MOF‐74‐l‐Pro material obtained in DMF, our studies show that l‐Pro is present as a defect‐capping structural element and not stuck within the pores. This is likely due to the presence of defects in the original MOF structure, that allow coordination of the amino acid molecule. Similarly, for the application of Zr‐MOFs in aldol asymmetric catalysis, the functionalisation with proline is achieved on defected MOFs, with reduced cluster connectivity.^[52]^ In the adsorption isotherm of the Zn‐MOF‐74‐l‐Pro synthesised in DMF, there is also a micropore to mesopore transition in the material after modification, shown by a hysteresis in the region 0.6–1.0 *p*/*p* ^0^ and the increased micropore diameter as compared to the pristine Zn‐MOF‐74 (Table S1). Indeed, both TGA and ^1^H NMR analysis also indicate that defects have been introduced in Zn‐MOF‐74 during the post‐synthetic modification, where dobdc^4−^ or Zn^2+^ are missing in the framework. In the material synthesised in MeOH there is no indication of defects, and a type I adsorption isotherm is obtained. This corresponds to a microporous structure, with a surface area of 264 m^2^ g^−1^, more than fourfold lower than Zn‐MOF‐74 (1168 m^2^ g^−1^). Zn‐MOF‐74‐l‐Pro samples synthesised in MeOH have little or no additional defects after PSM as confirmed by the size of the pore diameter (Table S1) and additional electron microscopy imaging (Figure S10).

**Scheme 1:**
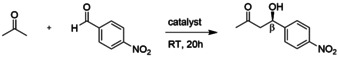
The asymmetric aldol reaction between acetone and 4‐nitrobenzaldehyde (pNBA) to obtain the chiral β‐aldol product. The reaction can also undergo water elimination to form the α,β‐unsaturated product.

**Figure 9:**
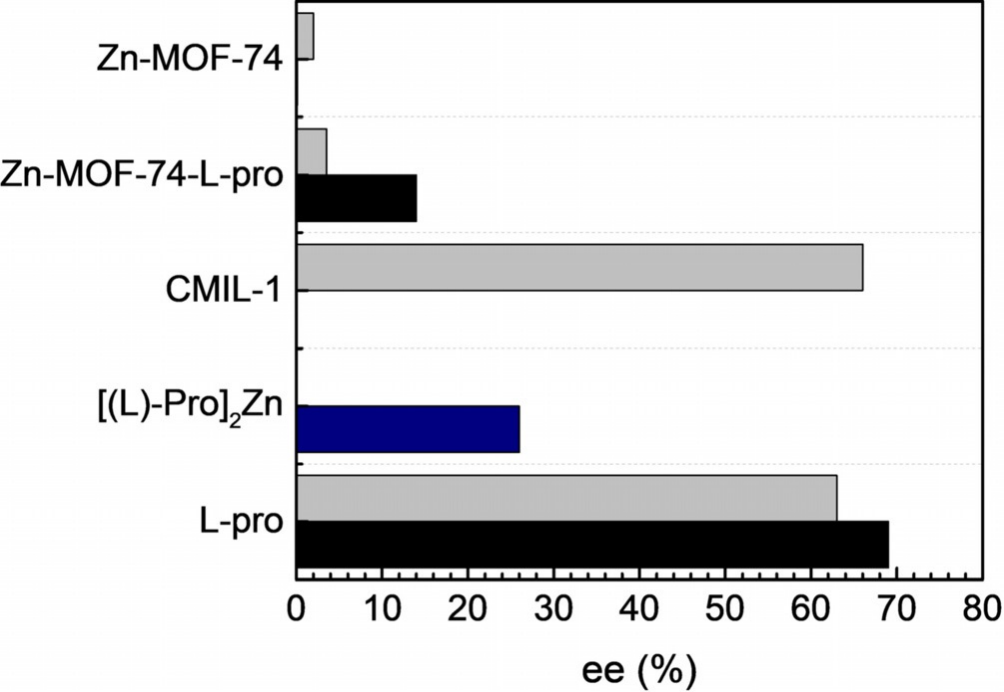
Enantioselectivity towards the (*R*)‐β‐aldol product in the asymmetric aldol reaction between acetone and pNBA by using 30 mol % loading of Zn‐MOF‐74‐l‐Pro in 4:1, *v*/*v*, THF/acetone (black) or DMF/acetone (grey). Literature reported *ee* values for using 10 mol % loading of CMIL‐1.^[27]^ 5 mol % loading of [Zn(l‐Pro)_2_] (blue) was tested in H_2_O.^[65]^

To prove the pore blocking effect, Zn‐MOF‐74‐l‐Pro was tested in the asymmetric aldol reaction between acetone and 4‐nitrobenzaldehyde (pNBA) (Scheme 1). The aldol reaction is usually catalysed by coordination compounds via preformed stable enolates, but also by organocatalysts through an enamine mechanism.^[62]^ In particular, l‐Pro is a well‐known asymmetric homogeneous catalyst for aldol reactions.[^63^, ^64^] It is reported that the [Zn(l‐Pro)_2_] complex is a water‐stable catalyst that catalyses enantioselectively the aldol reaction.^[65]^ Therefore, the choice of reactants, along with their corresponding β‐aldol product, is based on their good fit within the Zn‐MOF‐74 pores. The catalytic tests were also used to confirm the presence of proline within the MOF‐74 pores and not at the surface of the MOFs prepared. If proline would have been attached at the MOF‐74 surface, the aldol reaction would proceed in an enantioselective manner because the framework itself is a mild Lewis acid that can only influence the rate of the reaction. The role of Lewis acid catalysts was previously investigated by addition of chloride salts (e.g., ZnCl_2_) to the l‐Pro‐catalysed aldol reaction of cyclohexanone and pNBA.^[66]^ This lead to improved conversion and stereoselectivity without influencing the formation of the preferred enantiomer.^[66]^ Indeed, the catalytic testing of the pristine MOF‐74 shows no significant *ee* values (Figure 9) similar to the use of MIL‐101, another MOF containing mild Lewis acid sites used as catalyst for the asymmetric aldol reaction between acetone and pNBA that gave no *ee*.^[27]^ As expected, upon blocking the catalyst's pores, the incorporated chiral amino acid is not accessible and thus the asymmetric aldol reaction does not occur enantioselectively.

Initially, Zn‐MOF‐74‐l‐Pro was synthesised and catalytically tested by using non‐activated Zn‐MOF‐74 in DMF/ac (4:1, *v*/*v*) because DMF is similar to DMSO, the solvent employed in the asymmetric aldol between acetone and pNBA by using l‐Pro.[^64^, ^67^] The choice of solvent is related to the common use of DMF in MOF synthesis and using as‐synthesised materials would reduce the number of steps for catalyst preparation. To confirm that the structure obtained was the same, the material was characterised by PXRD and TGA (Figure S11). Indeed, the same PXRD pattern was obtained when compared to that of the material modified in DMF by using MOF‐74 with exposed open metal sites (Figure 4). Thus, the activation step does not influence the formation of the defected structure observed previously for the chiral Zn‐MOF‐74‐l‐Pro prepared in DMF. Obviously, the catalytic testing of this material in the asymmetric aldol reaction as a model reaction shows very low *ee* (Figure 9) due to blocking of the pores. A previous study of the same aldol reaction in DMF catalysed by CMIL‐1, a modified MIL‐101 with proline‐based ligands coordinated to the open chromium metal sites, shows fair enantioselectivity of 66 %, after 36 h (Figure 9).

To gain insight in the solvent effect in the post‐synthetic modification with proline, another polar aprotic solvent was chosen, that is, THF, which has a lower binding strength than DMF. The synthesis of Zn‐MOF‐74‐l‐Pro was performed in THF/acetone (4:1, *v*/*v*) and the resulting material characterised by using PXRD and TGA (Figure S13). PXRD analysis shows the incipient formation of the peak at 2 *θ*=9.2°, which is very pronounced in the case of Zn‐MOF‐74‐l‐Pro materials prepared in DMF. The reduced intensity of this peak for the Zn‐MOF‐74‐l‐Pro in THF indicates that the structure does not suffer major structural changes. Also the ca. 13 % initial weight loss around 100 °C on the TGA curve indicates that this material is a good candidate for catalysis, with more accessible active sites. (Figure S11). Indeed, the testing of the catalyst, in THF, shows improved *ee*. This can be attributed to the catalytic activity of proline molecules incorporated in the modified MOF, and not to the freely proline in solution which gives almost 70 % *ee* (Figure 9). Unlike our catalyst, in the presence of CMIL‐1, the reaction in THF does not occur, even after 48 h.^[27]^ This can possibly be attributed to the differences between CMIL‐1 and Zn‐MOF‐74‐l‐Pro, such as the proline binding within the framework.

In order to obtain a fair comparison to the CMIL‐1 catalyst, the subsequent catalytic tests of Zn‐MOF‐74‐l‐Pro were done using 10 mol % loading. The study focused on the materials synthesised in MeOH using activated MOF‐74 starting materials and the catalytic testing was done in DMF, THF, ACN and neat acetone (Figure S12). The pore blocking effect for these catalysts should not be observed as the pores of these catalysts are more accessible according to the porosity studies (Figure 8 and Table S1). The homogeneous catalyst was initially tested and showed moderate to good performances in all solvents, with the highest enantioselectivity in DMF. However, the heterogeneous proline modified MOF‐74 catalysts yielded less than 5 % of the β‐aldol product (Figure S12). We attribute the low performance of the catalyst to the low amount of proline present in the catalyst. It should not be related to pore accessibility since the integrity of the framework is retained after reaction as confirmed by FTIR and PXRD studies (Figures S13 and S14).

## Conclusions

The incorporation of proline within the Zn‐MOF‐74 framework was achieved by post‐synthetic modification. Combined experimental and theoretical studies show that this synthetic approach enables the introduction of both chirality and defects in the MOF framework. Vibrational circular dichroism studies demonstrate not only the binding of the l‐Pro to the Zn‐MOF framework but also that l‐Pro retains its chirality upon its incorporation within the MOF framework. The monodentate coordination of l‐Pro was determined by calculating the binding energies of proline with the Zn^2+^ metal ions, which was also confirmed by the experimental studies. The presence of defects as well as the strong coordination of proline and DMF to the Zn^2+^ within the framework reduces pore's accessibility, as indicated by the nitrogen adsorption studies and the low enantioselectivity obtained in the aldol reaction between acetone and 4‐nitrobenzaldehyde. To the best of our knowledge, this is the first study reporting on the facile synthesis of MOF‐74 derivatives containing both chiral centres and defects. Despite the low catalytic activity of the synthesised materials, we believe that this study has brought further insight on the potential of PSM as a general and effective method for the synthesis of chiral MOFs.

## Experimental Section

**Materials and synthesis**: All chemicals were purchased from Sigma Aldrich, except *l*‐ and d‐Proline which were purchased from TCI Chemicals, and used without further purification. The digestion of the MOF samples was performed following a reported procedure.^[47]^

**Zn‐MOF‐74**: Sample was prepared using a reported procedure^[68]^ and activated following a reported procedure.^[54]^ The MOF was heated under vacuum from room temperature to 80 °C, from 80 °C to 100 °C, from 100 °C to 150 °C, from 150 °C to 200 °C, and from 200 °C to 265 °C, at a constant rate of 4 °C min^−1^, with the temperature held at 1 h at the end of each ramp; except for at 265 °C, at for the temperature was held for 12 h. The same procedure was used to treat all MOFs prior to N_2_ adsorption measurements. The elemental composition found: C, 24.85; H, 2.78; Zn: 34.33.

**Zn‐MOF‐74‐d,l‐Pro (MeOH or DMF)**: The activated Zn‐MOF‐74 (0.029 g; 0.089 mmol; 1.0 equiv.) and d‐ or l‐Pro (0.010 g; 0.089 mmol; 1.0 equiv.) were added to a glass vial containing 2.5 mL methanol or DMF, resulting in a yellow‐white nonhomogeneous mixture. The vial was tightly closed and the reaction mixture was mixed using a roller bank with 80 rpm at RT for 48 h. The solid material was then centrifuged (4000 rpm, 10 min) and the supernatant was removed using a Pasteur pipette. The solid was collected and washed two times with fresh MeOH, then dried overnight at 70 °C yielding a yellow‐green crystalline material. For the Zn‐MOF‐74‐Pro samples obtained in MeOH, the elemental composition found for Zn‐MOF‐74‐l‐Pro was: C, 21.35; H, 3.67; N: 0.11. For Zn‐MOF‐74‐d‐Pro: C, 21.05; H, 3.75; N: 0.12. For Zn‐MOF‐74‐Pro materials synthesised in DMF, elemental composition found for Zn‐MOF‐74‐l‐Pro was: C, 33.85; H,4.45; N: 5.71. For Zn‐MOF‐74‐d‐Pro: C, 33.73; H,4.34; N: 5.78. Note that the elemental composition is included to further confirm the post‐synthetic modification of Zn‐MOF‐74 in DMF. Due to the presence of defects in the original and post‐synthesis modified material, it is not possible to propose a clear molecular formula.

**General method for the catalytic testing**: Each catalytic reaction was performed by mixing 4‐nitrobenzaldehylde (0.076 gram; 0.5 mmol; 1.0 equiv.), proline (0.017 gram; 0.15 mmol; 30.0 mol %) and acetone (1 mL; 13.61 mmol; 27.2 equiv.) in 4 mL of organic solvent, except for the neat testing. The reaction was carried out at room temperature. After 20 hours of reaction time, the reaction mixture was quenched with an aqueous saturated ammonium chloride solution (5 mL), the catalyst was removed by gravitational filtration and the reaction mixture was extracted with ethyl acetate (3×10 mL). The combined organic fractions were dried on magnesium sulphate, which was separated from the reaction mixture by gravitational filtration. Solvent evaporation of the filtrate followed by drying under vacuum at temperatures up to 100 °C resulted in a solid. This solid was dissolved in chloroform‐d and separated by TLC‐chromatography eluting with hexanes/ethyl acetate (3:1). The target compound was scraped off the support and eluted with ethanol using a microcolumn. The *ee* was determined in duplo by chiral‐phase HPLC analysis on a YMC column.

**Physical characterisation**: Infrared spectra (4000‐400 cm^−1^, resol. 1 cm^−1^) were recorded on a Varian 660 FTIR spectrometer using a KBr module. VCD spectra of the rotated solid samples, prepared as KBr pellets, were obtained on a Bruker Vertex 70 FTIR spectrometer equipped with a Bruker PMA50 VCD module. Powder X‐ray diffraction (3–60°, 2.5° min^−1^) measurements were carried out on a Rigaku Miniflex X‐ray Diffractometer using Cu Kα radiation (*λ*=1.5406 Å). Thermogravimetric analysis (35–500°, 5 K min^−1^) and differential scanning calorimetry were performed using a NETZSCH Jupiter ® STA 449F3 instrument. The measurements were carried out under flow of air (10 mL min^−1^) and protective argon (10 mL min^−1^). N_2_ adsorption isotherms were measured at 77° K on a Thermo Scientific Surfer instrument after stepwise evacuation from room temperature to 80 °C; from 80 °C to 100 °C; from 100 °C to 150 °C; from 150 °C to 200 °C, and from 200 °C to 265 °C at a constant rate of 4 °C min^−1^, with the temperature held for 1 h at the end of each ramp, except for at 265 °C, at which all samples were held for 12 h. 1H NMR spectra were recorded with Bruker Bruker AMX 400.1 MHz spectrometer. DMSO was used as solvent, and the 1H NMR spectra were referenced to the residual solvent signal. The morphology of the samples with sputtered gold was studied by using Scanning Electron Microscopy (SEM, FEI Verios 460 scanning electron microscope) operated at 5 kV. Solid‐state NMR spectra were recorded using a Bruker Avance III spectrometer equipped with a wide‐bore 9.4 T superconducting magnet (at Larmor frequencies of 400.13 and 100.9 MHz for ^1^H and ^13^C, respectively). Samples were packed into standard 4 mm magic angle spinning (MAS) rotors and spectra were recorded with a MAS rate of 12.5 kHz. ^13^C NMR spectra were recorded with cross polarisation (CP) from ^1^H using a contact pulse (ramped for ^1^H) of 1 ms and high‐power (ν_1_ ≈100 kHz) TPPM‐15 ^1^H decoupling was applied during acquisition. Signal averaging was carried out for 2048 transients with a recycle interval of 3 s. Chemical shifts are reported in ppm relative to TMS using *l*‐alanine (CH_3_ *δ*=20.5 ppm) as a secondary solid reference.

**Computational details**: High quality optimisations based on periodic unit cells using VASP 5.4.1 (parameters: ENCUT=500, PREC=High, LAPSH=.true, EDIFF=1e‐7, EDIFFG=‐2E‐3).[^69^, ^70^, ^71^, ^72^] The used functional SCAN + rVV10^[73]^ is a versatile van der Waals density functional developed by combining the Strongly Constrained Appropriately Normed (SCAN) *meta*‐GGA semi‐local exchange‐correlation functional with the rVV10 non‐local correlation functional.[^74^, ^75^] SCAN is comparable to hybrid functionals at the cost of about only 3 times PBE.

## Conflict of interest

The authors declare no conflict of interest.

## Supporting information

As a service to our authors and readers, this journal provides supporting information supplied by the authors. Such materials are peer reviewed and may be re‐organized for online delivery, but are not copy‐edited or typeset. Technical support issues arising from supporting information (other than missing files) should be addressed to the authors.

Supplementary
