# Supplementary material for: Synthesis of Chiral MOF‐74 Frameworks by Post‐Synthetic Modification by Using an Amino Acid
Source: Chemistry. 2020 Oct 20;26(61):13957–65. doi: 10.1002/chem.202002293 (PMC7702064; doi:10.1002/chem.202002293)
Supplement: Supplementary file 1 — Supplementary [file CHEM-26-13957-s001.pdf]

Chemistry–A European Journal

Supporting Information

# Synthesis of Chiral MOF-74 Frameworks by Post-Synthetic Modification by Using an Amino Acid

Andreea Gheorghe,[a] Benjamin Strudwick,[a, b] Daniel M. Dawson,[c] Sharon E. Ashbrook,[c] Sander Woutersen,[a] David Dubbeldam,[a] and Stefania Tanase*[a]

# Supplementary information

## Synthesis of chiral MOF-74 frameworks using post-synthetic modification by an amino acid

Andreea Gheorghe,[a] Benjamin Strudwick,[a,b] Daniel M. Dawson,[c] Sharon E. Ashbrook,[c] Sander Woutersen,[a] David Dubbeldam[a] and Stefania Tanase*[a]

[a.] Van 't Hoff Institute for Molecular Sciences, University of Amsterdam, Science Park 904, 1098 XH, Amsterdam, The Netherlands.

[b.] Current address: Paul Scherrer Institute, ETH Zürich, Forschungsstrasse 111, 5232 Villigen PSI, Zürich, Switzerland.

[c.] EaStCHEM School of Chemistry and Centre of Magnetic Resonance, University of St Andrews, North Haugh, KY16 9ST, St Andrews United Kingdom.

*To whom correspondence should be addressed.

E-mail: s.grecea@uva.nl

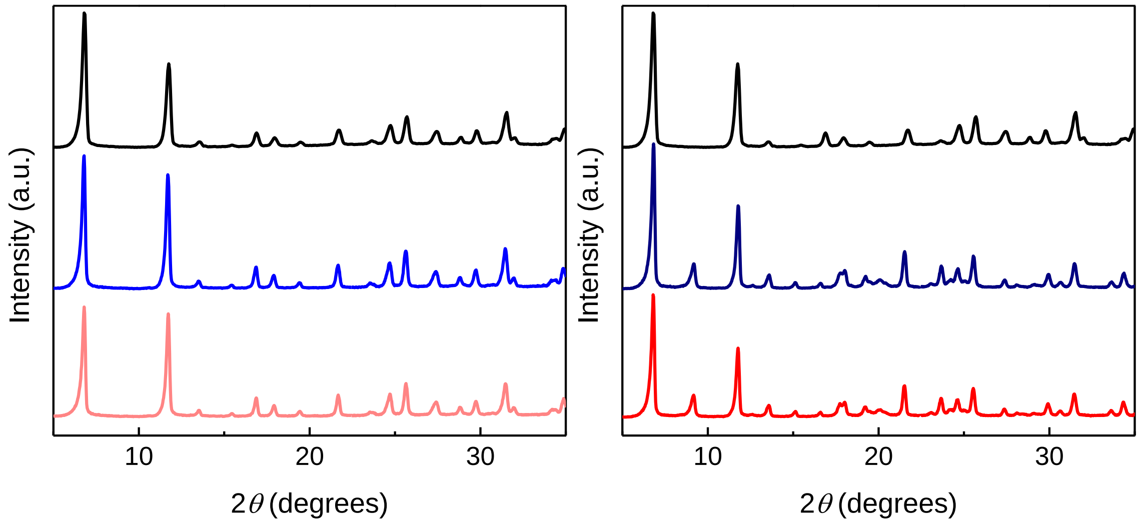


**Figure S1**. PXRD patterns of Zn-MOF-74 (black), MOFs synthesised in MeOH with *L*-Pro (blue) and with *D*-Pro (red) (left) and MOFs synthesised in DMF with *L*-Pro (red) and with *D*-Pro (blue) (right).

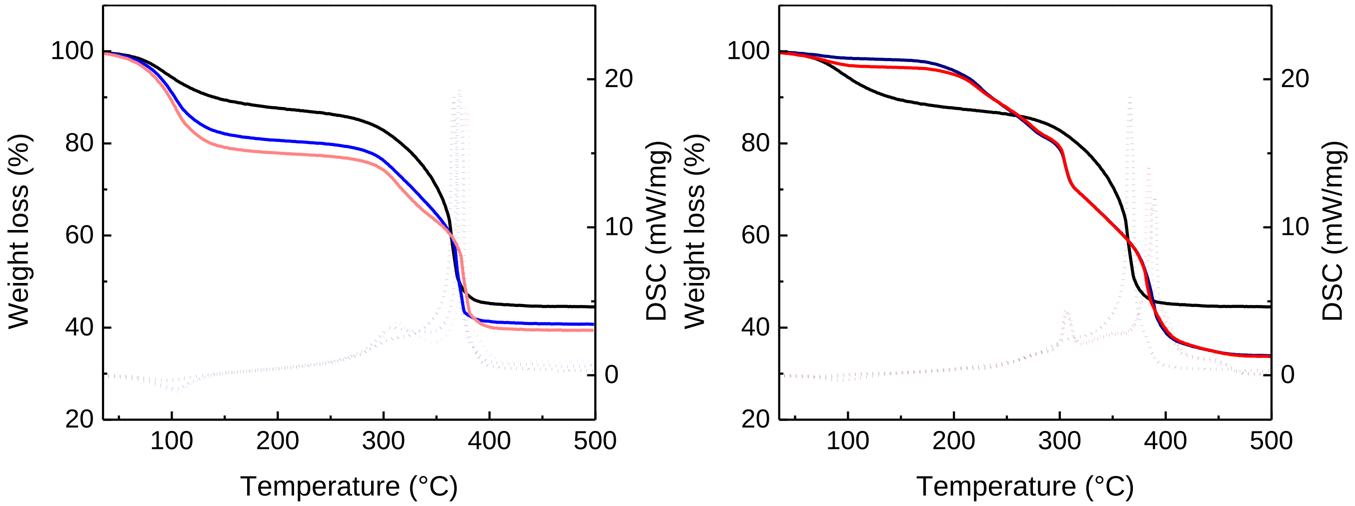


**Figure S2**. TGA/DSC curves of Zn-MOF-74 (black), MOFs synthesised in MeOH with *L*-Pro (blue) and with *D*-Pro (red) (left) and MOFs synthesised in DMF with *L*-Pro (red) and with *D*-Pro (blue) (right).

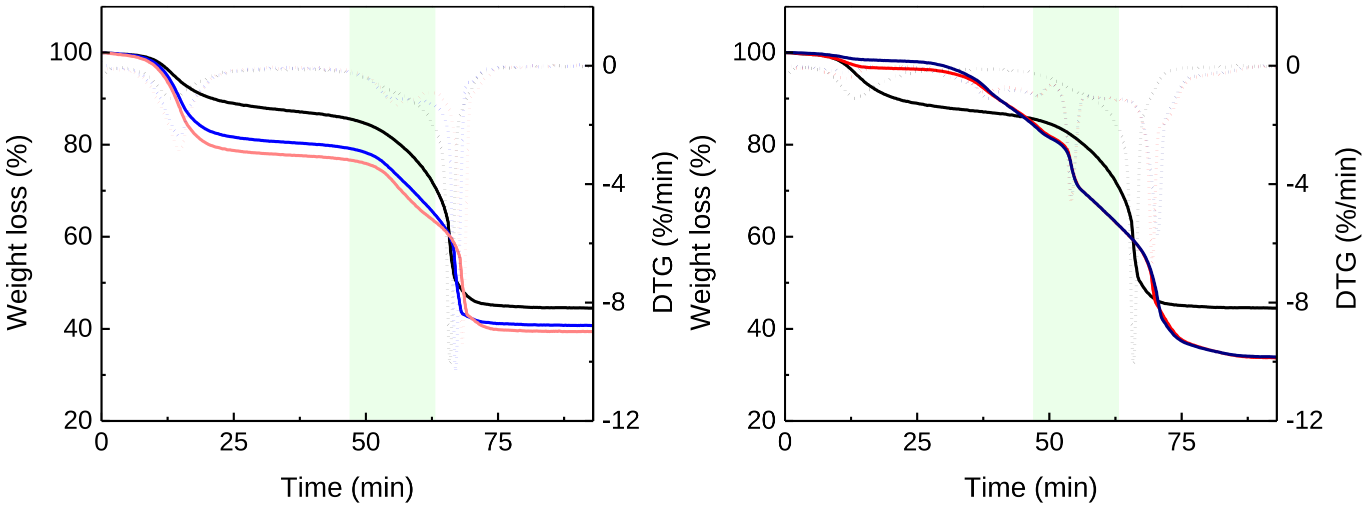


**Figure S3**. TGA/DTG curves of Zn-MOF-74 (black), MOFs synthesised in MeOH with *L*-Pro (blue) and with *D*-Pro (red) (left) and MOFs synthesised in DMF with *L*-Pro (red) and with *D*-Pro (blue) (right).

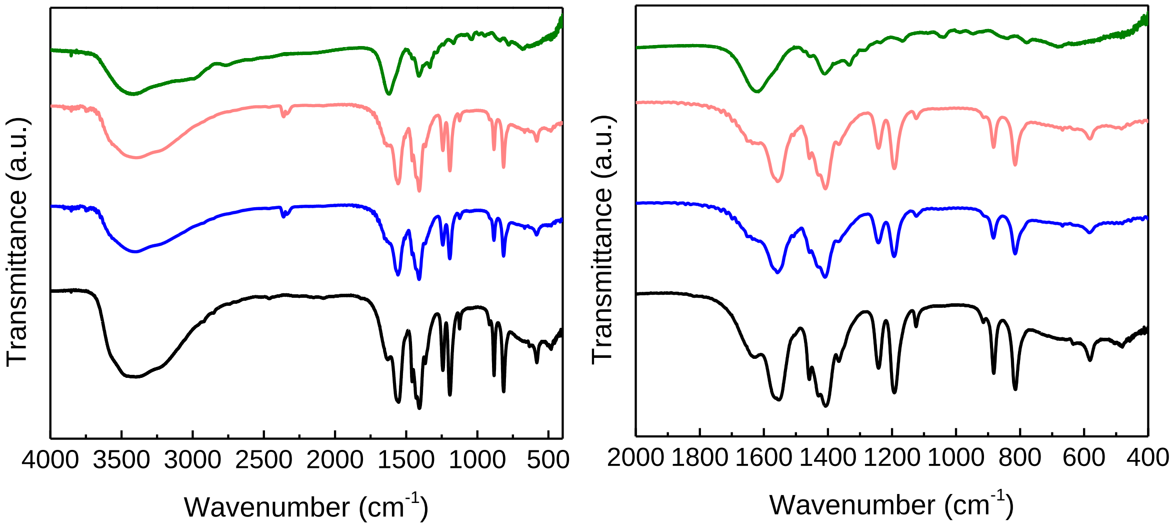


**Figure S4**. FTIR spectra of *L*-Pro (green), Zn-MOF-74 (black), MOFs synthesised in MeOH with *L*-Pro (blue) and with *D*-Pro (red) (left) and FTIR with zoom in on the fingerprint region (right).

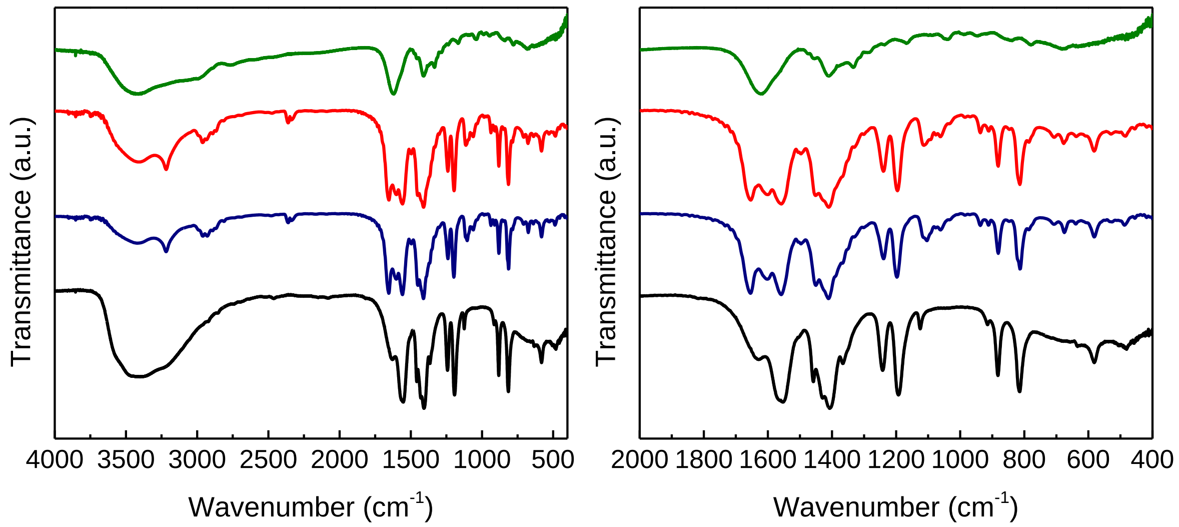


**Figure S5**. FTIR spectra of *L*-Pro (purple), Zn-MOF-74 (black), MOFs synthesised in DMF with *L*-Pro (red) and with *D*-Pro (blue) (left) and FTIR with zoom in on the fingerprint region (right).

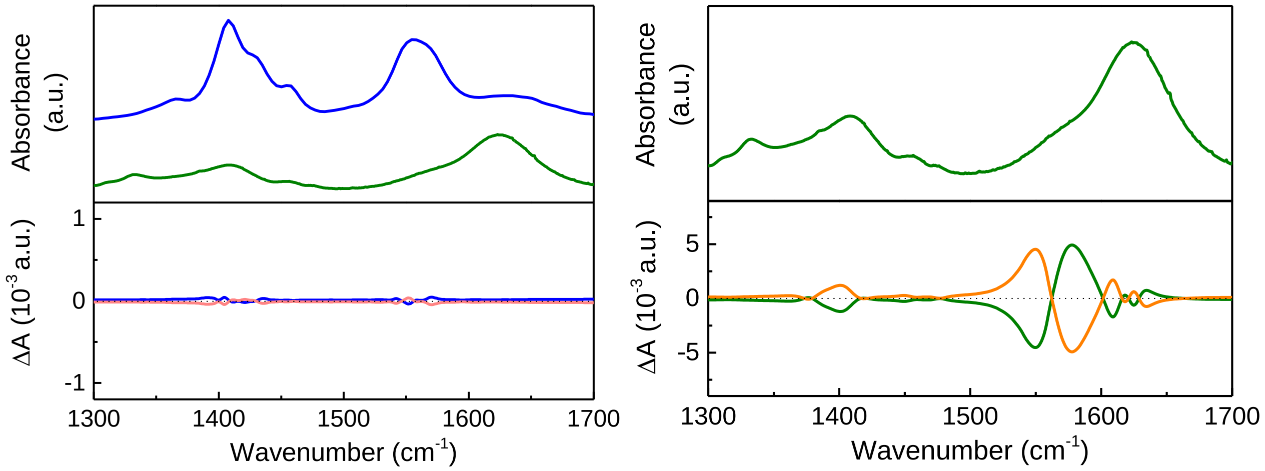


**Figure S6**. Left: FTIR spectra of *L*-Pro (green), MOFs synthesised with *L*-Pro in MeOH (blue) (top) and refined VCD spectra of MOFs synthesised in MeOH with *L*-Pro (blue) and with *D*-Pro (red) (bottom). Right: FTIR spectra of *L*-Pro (green) (top) and refined VCD spectra of *L*-Pro (green) and D-Pro (orange) (bottom) (right).

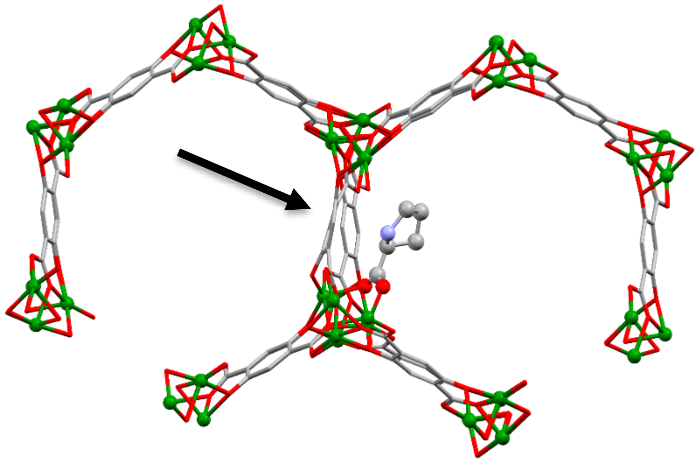

**Figure S7**. View along the c axis of the MOF-74 framework with induced strain by coordination of one proline molecule to two consecutive $Zn^{2+}$ ions in a bidentate fashion.

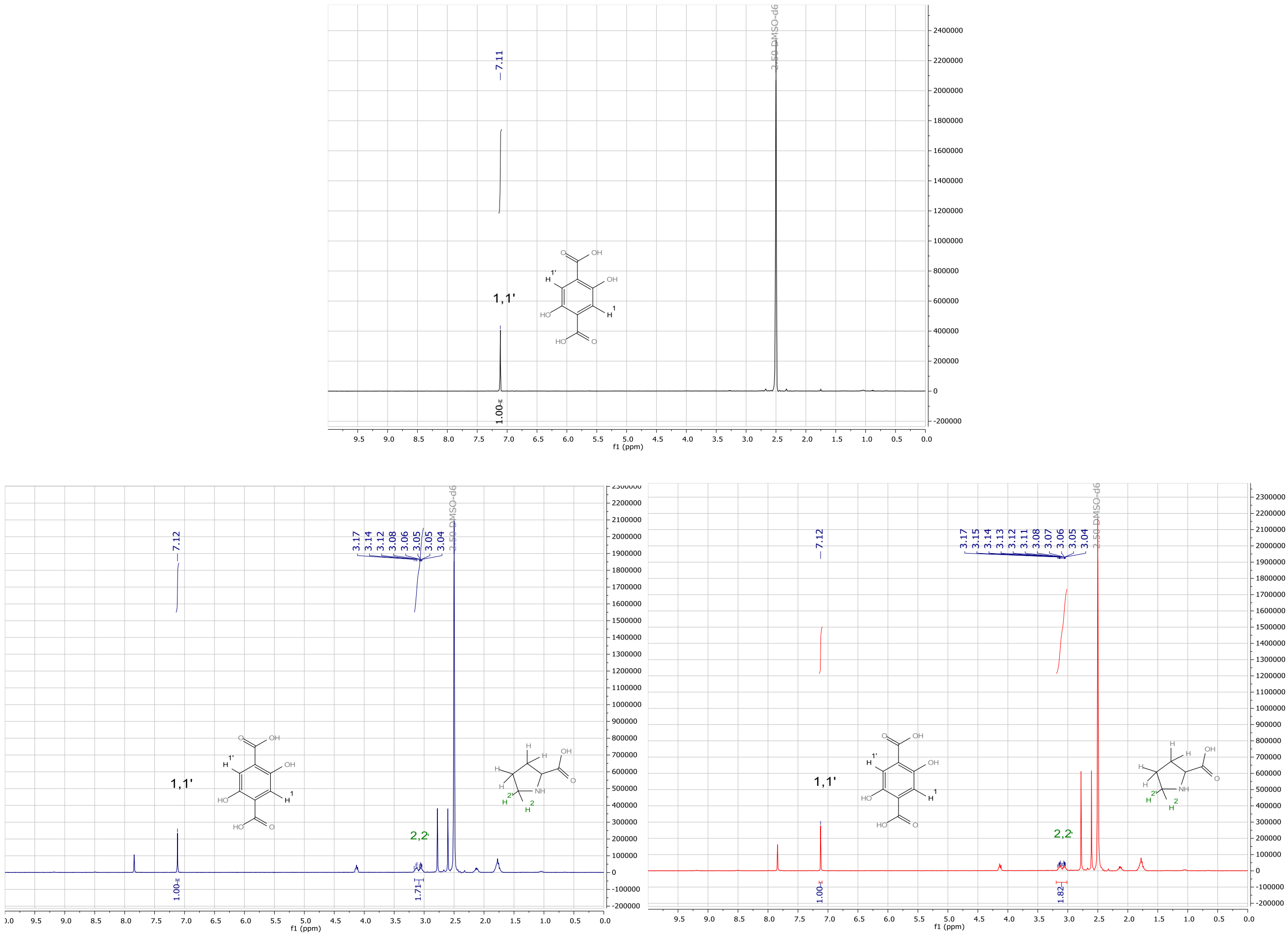


**Figure S8**. $^{1}H$ NMR analysis of the digested Zn-MOF-74 (black) and of the MOFs synthesised in DMF with *D*-Pro (blue) (left) and with *L*-Pro (red) (right).

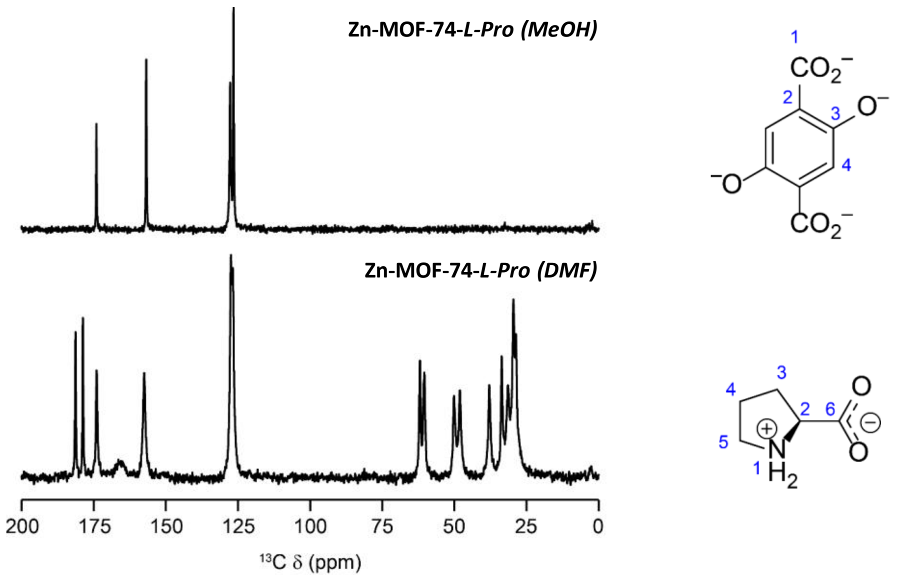


**Figure S9**. $^{13}C$ CP MAS NMR spectra of Zn-MOF-74-*L-Pro* synthesised in MeOH and DMF and the numbering used for dobdc$^{4-}$ and *L-Pro*.

**Table S1**. Textural values of Zn-MOF-74 and Zn-MOF-74-*L-Pro* synthesised in MeOH and DMF.

| Sample | $S_{\text{Langmuir}}$ ($m^2\ g^{-1}$) | $D_{\text{micropore}}$ (nm) | $V_{micropore}{}^{a}$ ($cm^3\ g^{-1}$) |
|---|---|---|---|
| Zn-MOF-74 | 1168 | 1.6 | 0.4 |
| Zn-MOF-74-*L*-Pro (MeOH) | 264 | 1.5 | 0.09 |
| Zn-MOF-74-*L*-Pro (DMF) | 37 | 2.0 | 0.02 |
| a. The Saito-Foley volume is calculated using the range $p/p^0$ 0.001-0.4. | | | |

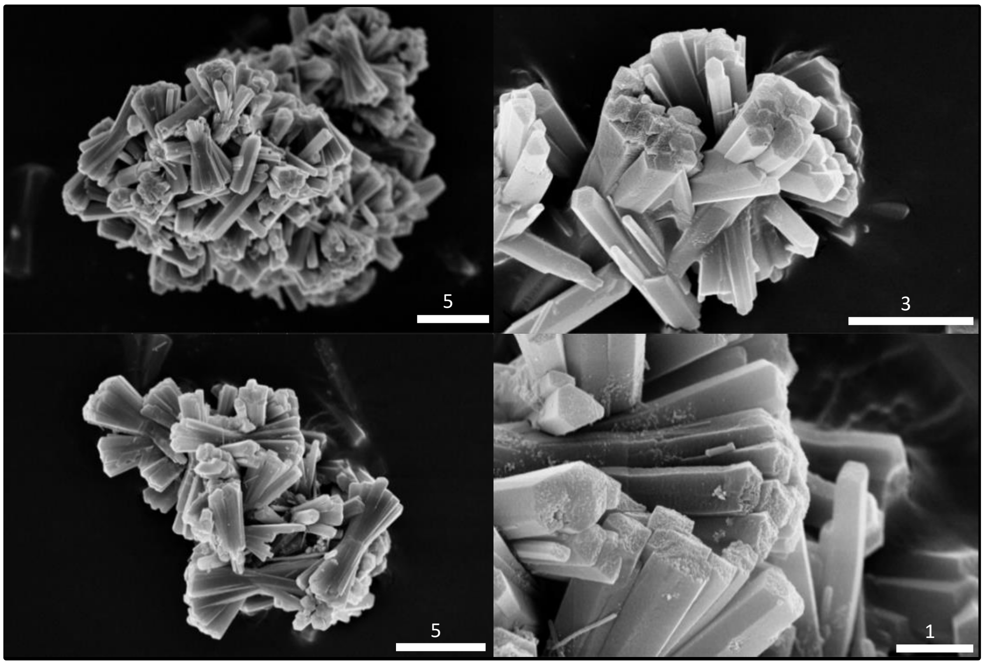


Figure S10. SEM image of MOFs synthesised in MeOH with *L*-Pro (top) and with *D*-Pro (bottom).

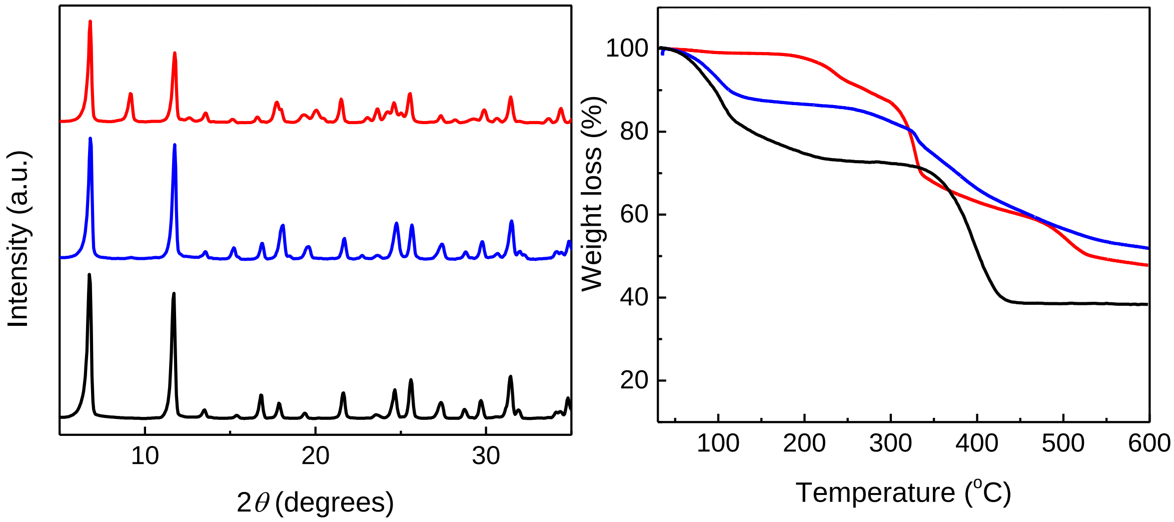


Figure S11. PXRD patterns (left) and TGA curves (right) of non-activated Zn-MOF-74 (black), Zn-MOF-74-*L*-Pro prepared in THF/ac (blue) and in DMF/ac (red). The TGA measurements were carried under inert conditions using a continuous 20 mL/min Ar flow, and a heating ramp of 10K/min.

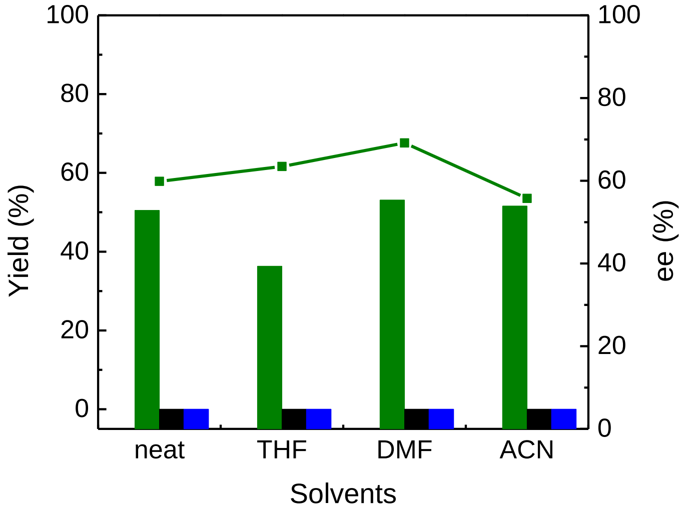

**Figure S12**. Catalytic results of the asymmetric aldol reaction of pNBA and acetone in different solvents. Reaction conditions: 0.5 mmol *para*-nitro-benzaldehyde, 10 mol % catalyst loading and 5 mL solvent/acetone 4/1 (v/v), 20 h reaction time at room temperature. Green line symbolises the *ee* values obtained when using *L*-Pro as catalyst which were calculated based on chiral HPLC chromatography. The column bars represent the yields when using *L*-Pro (green), MOF-74 (black) or Zn-MOF-74-*L*-Pro (blue) and were calculated based on $^{1}H$ NMR spectra.

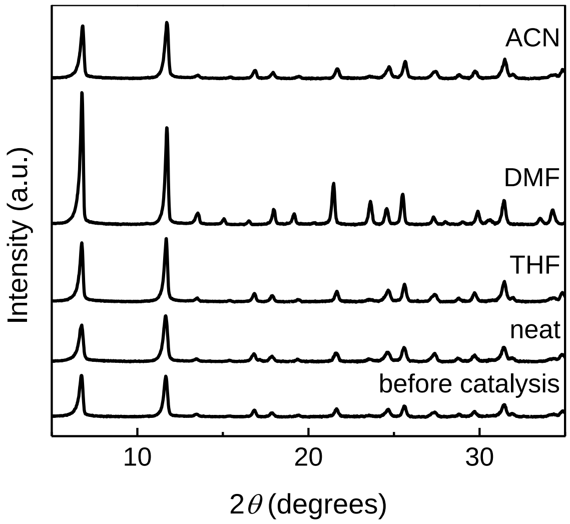

Figure S13. PXRD patterns of Zn-MOF-74-*L*-Pro catalysts before and after testing in the aldol reaction.

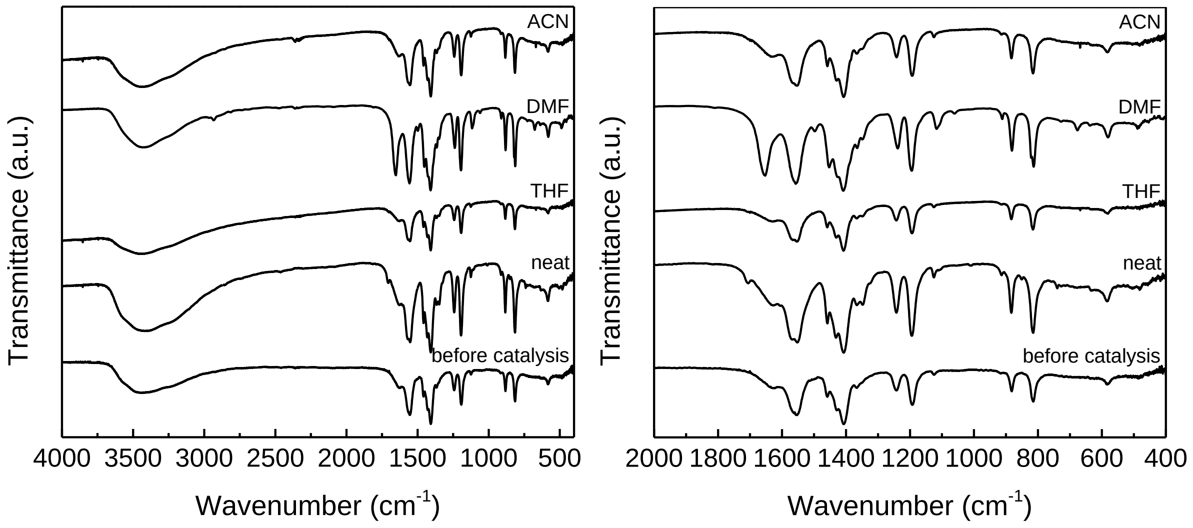


Figure S14. FTIR spectra of the Zn-MOF-74-*L*-Pro catalyst before and after testing in the aldol reaction (left) and FTIR with zoom in on the fingerprint region (right).

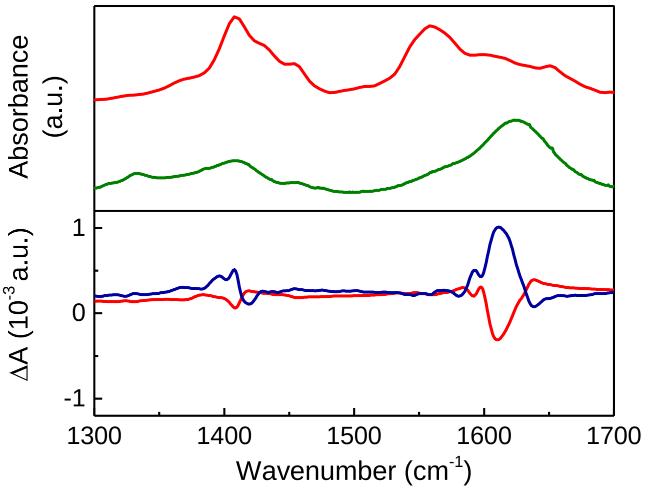


Figure S15. FTIR spectra of *L*-Pro (green), MOFs synthesised with *L*-Pro in DMF (red) (top) and raw VCD spectra of MOFs synthesised in DMF with *L*-Pro (red) and with *D*-Pro (blue) (bottom).

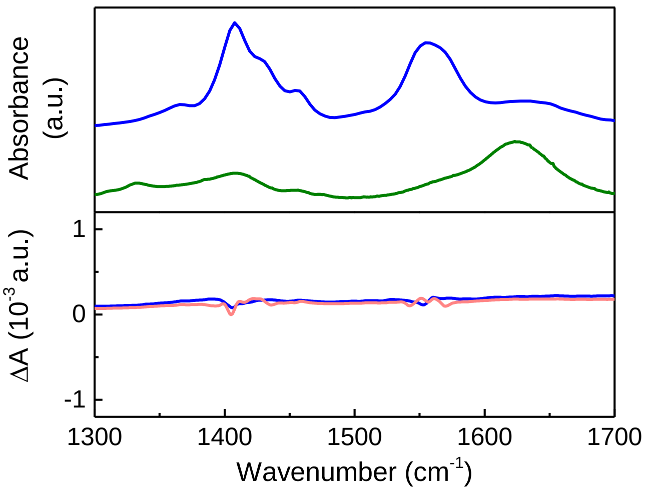


Figure S16. FTIR spectra of *L*-Pro (green), MOFs synthesised with *L*-Pro in MeOH (blue) (top) and raw VCD spectra of MOFs synthesised in MeOH with *L*-Pro (blue) and with *D*-Pro (red) (bottom).

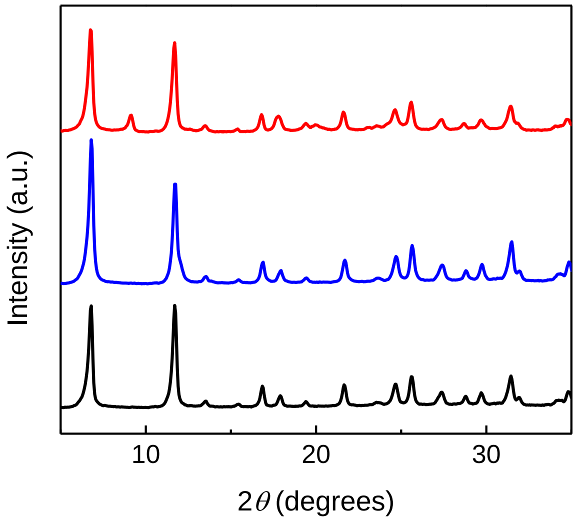


Figure S17. PXRD patterns of Zn-MOF-74 (black), Zn-MOF-74-*L-Pro* synthesised in MeOH (blue) and in DMF (red) after nitrogen adsorption studies.

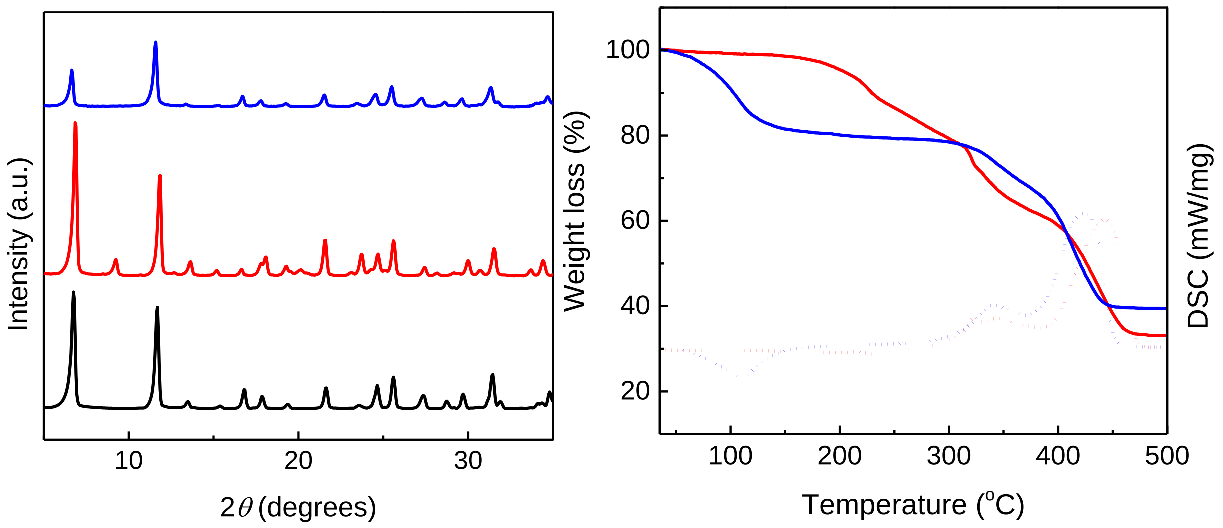


Figure S18. PXRD patterns (left) and TGA-DSC (right) of Zn-MOF-74 (black), Zn-MOF-74-*L-Pro* synthesised in MeOH (blue) and in DMF (red) using a molar ratio of 1:2 *L-Pro* to Zn-MOF-74. The TGA-DSC analysis was performed with a heating rate of 10 °Cmin$^{-1}$ and 20 mLmin$^{-1}$ flow of Ar.
